# Probiotic characteristics and zearalenone-removal ability of a *Bacillus licheniformis* strain

**DOI:** 10.1371/journal.pone.0194866

**Published:** 2018-04-11

**Authors:** Tsui-Chun Hsu, Ping-Jung Yi, Ting-Yu Lee, Je-Ruei Liu

**Affiliations:** 1 Department of Animal Science and Technology, National Taiwan University, Taipei, Taiwan; 2 Institute of Biotechnology, National Taiwan University, Taipei, Taiwan; 3 Agricultural Biotechnology Research Center, Academia Sinica, Taipei, Taiwan; LAAS-CNRS, FRANCE

## Abstract

Zearalenone (ZEN) is a mycotoxin produced by *Fusarium* species, which is one of the main animal feed contaminants causing reproductive disorders in livestock. The aim of this study was to evaluate the probiotic characteristics and ZEN removal ability of a *Bacillus licheniformis* strain CK1. The probiotic properties, including acidic tolerance, bile salt tolerance, adherence capability, and anti-pathogenic activities of CK1 were evaluated. CK1 survived after incubation at pH 2.0 or 3.0 for 3 h, grew well in LB broth containing 0.3% oxgall, possessed adherence capability to Caco-2 cells, and inhibited the growth of *Escherichia coli* O157:H7 and *Listeria monocytogenes*. The ZEN removal ability of CK1 was compared with a mineral mycotoxin-adsorbing agent, hydrated sodium calcium aluminosilicate (HSCAS), and a well-characterized biological mycotoxin-adsorbing agent, *Lactobacillus rhamnosus* GG (LGG). At 37°C in phosphate-buffered saline (PBS, pH 7.0) containing 5 μg mL^-1^ of ZEN, the ZEN removal percentage of CK1 was 73.0%, which was significantly higher than that of HSCAS and LGG (45.9% and 48.4%, respectively). In the pH range of 2.5–8.0, CK1 removed up to 65% of ZEN. At temperatures between 4 and 42°C, CK1 removed more than 75% of ZEN. In the adsorption stability analysis, the amounts of ZEN removed by CK1 was over 30% even after five consecutive rounds of washing procedures. These findings demonstrated that CK1 displayed probiotic characteristics and removed ZEN effectively. Therefore, CK1 has a great potential for the development of feed additive to remove ZEN.

## Introduction

Zearalenone (ZEN) is a mycotoxin produced by various species of *Fusarium*, and frequently occurs in maize, barley, oats, wheat, and sorghum [1,2]. A survey from 2009 to 2011 on the occurrence of ZEN in feedstuffs and feed showed that ZEN was detected at an average level of 104 ppb, with a 45% contamination rate in 5,402 corn, soybean, soybean meal, wheat, dried distillers grains with solubles, and finished feed samples from Americas, Europe and Asia [3]. Another survey from 2004 to 2011 on the occurrence of ZEN in feed and feed raw materials showed that ZEN was detected at an average level of 101 ppb, with a 36% contamination rate in 13,578 corn, soybean, soybean meal, wheat, barley, dried distillers grains with solubles, silage, and finished feed samples from all over the world [4]. ZEN is one of the primary animal feed contaminants causing reproductive disorders in livestock. ZEN competes with cytosolic estrogen receptors present in the uterus and mammary glands via its estrogenic properties [5,6]. A previous study showed that the concentrations of 1 to 5 μg of ZEN per gram of feed resulted in the clinical symptoms in swine, such as enlargement of the vulva, hypertrophy of the mammary glands, and occasional prolapse of the uterus in severe cases [7]. Several strategies have been developed to decontaminate and/or detoxify mycotoxin in the mycotoxin-contaminated feed products. Two specialized feed additives are used to prevent mycotoxicosis in animals: mycotoxin-biotransforming agents and mycotoxin-adsorbing agents [8].

The biotransforming agents, such as bacteria, yeast, fungi, and enzymes, convert ZEN into less- or non-toxic metabolites [9–17]. Despite some publications claiming that many microorganisms possess ZEN biotransforming abilities, their applications in practice in detoxification of food and feed are limited. Biotransformation of ZEN does not represent detoxification because it can yield even stronger estrogenic cognates such as α-zearalanol and β-zearalanol [18]. In addition, a lack of information about mechanisms of biodegradation, toxicity of degradation products, and safety of the microorganisms in animals limit the application of ZEN-biotransforming agents for use in animal feed.

Inactivation of mycotoxins by adsorbing agents has been extensively reviewed elsewhere [19–24]. There are two types of mycotoxin-adsorbing agents: mineral and biological mycotoxin-adsorbing agents. The most commonly used mineral mycotoxin-adsorbing agents are aluminosilicates, such as bentonites, clinoptilolites, montmorillonites, hydrated sodium calcium aluminosilicate (HSCAS), and zeolites [25,26]. HSCAS is the most studied mycotoxin-adsorbing agent and is characterized as a good aflatoxin-adsorbing agent. However, its efficacy against other mycotoxins is very limited [27]. On the other hand, some biological mycotoxin-adsorbing agents such as yeast cell walls derived from *Saccharomyces cerevisiae* and some lactic acid bacterial strains such as *Lactobacillus rhamnosus* GG (LGG), *L*. *rhamnosus* LC-705, *L*. *acidophilus*, and *L*. *plantarum* are more welcomed because of their wider range of mycotoxin adsorption abilities [15,19,28–30].

In a previous study, *Bacillus licheniformis* strain CK1 was isolated from a soil sample and demonstrated that it possessed ZEN removal ability [31]. Furthermore, CK1 could detoxify ZEA in feed and reduce the adverse effects of ZEA in the gilts [32]. The aim of this study was to evaluate the probiotic characteristics and ZEN removal ability of CK1. We evaluated the probiotic properties, including acid and bile salt tolerance, adherence capability, and anti-pathogenic activities of CK1. We also evaluated the ZEN removal ability of CK1 by comparing CK1 with a mineral mycotoxin-adsorbing agent, HSCAS, and a well-characterized biological mycotoxin-adsorbing agent, LGG. In addition, the effects of pH and temperature on the ZEN-removal ability of CK1 and the ZEN adsorption stability of CK1 were investigated.

## Materials and methods

### Chemicals and reagents

HSCAS was provided by Bestar Laboratories LTD (Taipei). ZEN powder was purchased from Sigma-Aldrich Co. (St. Louis, MO) and was dissolved in methanol (1 mg mL^-1^) to prepare a stock solution and stored in the dark at -20°C and brought to room temperature before use. The ZEN standard solution for high-performance liquid chromatography (HPLC) calibration or spiking purposes was purchased from Sigma-Aldrich Co. Acetonitrile and methanol (HPLC grade) were supplied by J. T. Baker Inc. (Phillipsburg, NJ). Water for the HPLC mobile phase was purified successively by reverse osmosis and a Milli-Q system (Millipore, Bedford, MA). All the other chemicals used in this study were of analytical reagent grade and were obtained from Sigma-Aldrich Co. All solutions prepared for HPLC were filtered through a 0.22 μm-pore size filter (Millipore) before use.

### Bacterial strains, cell line, and culture conditions

CK1 (DSM 025954) was isolated and identified as previously demonstrated [31] while LGG (ATCC 53013) was obtained from the American Type Culture Collection (ATCC; Manassas, VA). CK1 was cultured in Luria-Bertani (LB) broth (Difco Laboratories, Detroit, MI) at 37°C for 16 h in an orbital shaker at 200 rpm (Major Science Inc., Taipei, Taiwan). LGG was cultured in de Mann, Rogosa, Sharpe (MRS) broth (Oxoid, UK) at 37°C for 16 h without shaking. The bacterial concentrations were measured by optical density readings at 600 nm or by traditional colony counting methods [33].

The pathogens used for the evaluation of anti-pathogenic activity of CK1 included *Bacillus cereus* ATCC 11778, *Escherichia coli* O157:H7 ATCC 35150, *Listeria monocytogenes* BCRC 15338, and *Salmonella enterica* subsp. *enterica* BCRC 12947. All the pathogens were purchased from the Bioresource Collection and Research Center (BCRC; Hsinchu, Taiwan) and cultured according to the instructions provided by BCRC. *Bacillus cereus* ATCC 11778, *Escherichia coli* O157:H7 ATCC 35150, and *Salmonella enterica* subsp. *enterica* BCRC 12947 were cultured in nutrient broth (Difco Laboratories) at 37°C with shaking at 250 rpm, while *Listeria monocytogenes* BCRC 15338 was cultured in brain heart infusion broth at 37°C with shaking at 250 rpm.

The cancer-derived human colonic intestinal epithelial cell line Caco-2 was used for the in vitro assay of bacterial adhesion to mammalian epithelial cells. The Caco-2 cells were purchased from BCRC (Hsinchu, Taiwan) and were routinely grown at 37°C under a humidified atmosphere of 95% air and 5% CO_2_ in Dulbecco’s Modified Eagle Medium (Biological Industries, Bet-Haemek, Israel) supplemented with 4 mM L-glutamine (Biological Industries), 1.5 g L^-1^ sodium bicarbonate (Biological Industries), 4.5 g L^-1^ glucose (Sigma-Aldrich Co., St Louis, MO), 10 mg L^-1^ human transferrin (Sigma-Aldrich Co.), and 10% fetal calf serum (Moregate Biotech, Queensland, Australia).

### Acidic tolerance of CK1

Acid tolerance of CK1 was determined according to the method described by Liu et al. [34]. A 1-mL aliquot of the 24-h culture of CK1 was centrifuged at 9,000×*g* for 10 min at 4°C (Tomy MX-301, Tomy Digital Biology Co., LTD, Tokyo, Japan). The resultant pellets were washed twice with sterile PBS (0.1 M, pH 7.0), and then resuspended in 10 mL of sterile PBS (0.1 M, pH 2.0 or 3.0). The bacterial suspensions were incubated at 37°C for 3 h in an orbital shaker at 200 rpm. During the incubation period, a 0.1-mL aliquot of sample was taken at 0, 30, 60, and 180 min respectively and plated onto LB agar to determine the total number of viable cells using the traditional colony counting methods.

### Bile salt tolerance of CK1

Bile salt tolerance of CK1 was determined as previously described by Liu et al. [34]. Briefly, a 1-mL aliquot of the 24-h culture of CK1 was inoculated into 100 mL of LB broth containing 0.3% (w/v) oxgall (Sigma-Aldrich Co.), and incubated statically at 37°C for 10 h. During the incubation period, a 1-mL aliquot of sample was taken at 0, 2, 4, 6, 8, and 10 h, respectively, to estimate the bacterial concentrations by measuring optical density at 600 nm.

### Adhesion capability of CK1

The Caco-2 cells were incubated with antibiotic-free medium at 37°C for 24 h and then removed from tissue culture flasks with an EDTA-trypsin solution. After washing three times with sterile PBS (0.1 M, pH 7.0), the Caco-2 cells were resuspended in PBS containing mouse anti-human CD29 (integrin beta 1) monoclonal antibody conjugated with PerCP-eFluor 710 (diluted 1:10 in PBS; eBioscience. Inc., San Diego, CA) and incubated at room temperature for 1.5 h. Then, the immunostained Caco-2 cells were washed three times with PBS to a concentration of 1 × 10^6^ cells mL^-1^.

The CK1 cells were cultivated, harvested, and resuspended in 1 mL of sterile PBS (0.1 M, pH 7.0) as described above and were then labeled with 5(6)-carboxyfluorescein diacetate N-succinimidyl ester (CFSE, Sigma-Aldrich Co.) as described by Hsueh et al. [35]. Fluorescently labeled bacterial cells were added into the Caco-2 suspension to yield a final concentration of 1 × 10^8^ CFU mL^-1^ of bacteria and 1 × 10^6^ cells mL^-1^ of Caco-2 cells. After incubating at 37°C for 2 h, the Caco-2 cells were harvested by centrifugation at 5,000×*g* for 20 min at 4°C and were washed three times with PBS to remove non-adherent bacteria. The confocal microscopic observation of the Caco-2 cells with adherent bacteria was performed using a Leica TCS SP5 II spectral confocal microscope mounted on a Leica DMI 6000B inverted microscope (Leica Microsystems, Heidelberg, Germany) with a HCX PLAPO CS 63×/1.4–0.6 oil immersion objective. In addition, the fluorescence intensity of the Caco-2 cells with adherent bacteria was measured using the Cytomics FC500 Flow Cytometry System (Beckman Coulter, Inc., Fullerton, CA) with a 488-nm argon laser and a 525-nm emission filter for CFSE detection and a 755-nm emission filter for PerCP-eFluor 710 detection. Flow cytometry was gated to include Caco-2 cells and to exclude cellular debris and non-adherent bacteria using a dot plot displaying forward scatter (FSC) vs. side scatter (SSC). Cells inside the gated area were confirmed by staining with CD29 antibody conjugated with PerCP-eFluor 710. For each analysis, 10,000 events were acquired and the flow cytometric data obtained were analyzed using CXP software (Beckman Coulter, Inc.) to produce the histograms of each individual particle sample and to calculate the mean red fluorescence intensity for each cell population.

### Anti-pathogenic activities of CK1

Anti-pathogenic activities of CK1 were analyzed by an agar plate diffusion method [36]. A 5-mL aliquot of the 24-h culture of CK1 was centrifuged at 9,000×*g* for 10 min at 4°C. The resultant supernatants were adjusted to pH 7.0, and then were sterilized by filtering through a 0.22-μm nylon filter. Then, 50 μL of the filtered neutralized supernatants were added to filter paper discs (6 mm diameter), which were placed on the agar plate surface previously inoculated with indicator pathogens including *B*. *cereus* ATCC 11778, *E*. *coli* O157:H7 ATCC 35150, *L*. *monocytogenes* BCRC 15338, and *S*. *enterica* subsp. *enterica* BCRC 12947. After incubation at 37°C for 24 h, the diameter of a clear zone around the discs was measured.

### Comparison of the ZEN removal abilities of HSCAS, LGG, and CK1

The ZEN removal abilities of HSCAS, LGG, and CK1 were determined according to the methods described by Ramos et al. [22] and El-Nezami et al. [29]. Briefly, a 0.1-mL aliquot of the 24-h culture of LGG and CK1 was inoculated into 10 mL of MRS and LB broth, respectively. After 24 h of incubated at 37°C, a 5-mL aliquot of the 24-h culture of CK1 and LGG was centrifuged at 9,000×*g* for 10 min at 4°C and the cell pellets were washed twice with sterile phosphate-buffered saline (PBS; 0.1 M, pH 7.0). Then, the HSCAS powders, CK1 cells, or LGG cells were added to 5 mL of PBS (0.1 M, pH 7.0) containing 5 μg mL^-1^ of ZEN to yield a final HSCAS concentration of 1% (w/v) or bacterial concentration of 1 × 10^10^ CFU mL^-1^. The matrix control and the substrate control were conducted with PBS without ZEN and PBS containing 5 μg mL^-1^ of ZEN but without adsorbing agents, respectively. Once the HSCAS powders or bacterial cells were added to the PBS containing ZEN, aliquots of 1 mL were taken immediately and centrifuged at 9,000×*g* for 10 min at 4°C, and the resultant supernatants were analyzed for ZEN concentration.

### Effect of acid and heat treatments on the ZEN removal abilities of CK1

The CK1 cells were cultivated and harvested as described above. The collected cell pellets were subjected to three different treatments: suspension in PBS (0.1 M, pH 7.0) and incubation at 37°C for 1 h, suspension in 2 M HCl solution and incubation at 37°C for 1 h, or suspension in PBS (0.1 M, pH 7.0) and autoclaving at 121°C for 15 min. After each treatment, the cells were harvested by centrifugation at 9,000×*g* for 10 min at 4°C and were washed three times with PBS (0.1 M, pH 7.0). The cells were then added to 5 mL of PBS (0.1 M, pH 7.0) containing 5 μg mL^-1^ of ZEN to yield a final concentration of 1 × 10^10^ CFU mL^-1^ of bacterial cells. Aliquots of 1 mL were taken immediately from the mixtures and centrifuged at 9,000×*g* for 10 min at 4°C, and the resultant supernatants were analyzed for ZEN concentration.

### Effect of ZEN concentrations on the ZEN removal ability of CK1

The heat-treated CK1 cells were prepared as described above and were added to 5 mL of PBS (0.1 M, pH 7.0) containing different concentrations of ZEN (0.5, 5, 50, and 200 μg mL^-1^) to yield a final concentration of 1 × 10^10^ CFU mL^-1^ of bacterial cells. The mixtures were incubated at 37°C for 24 h on an orbital shaker at 120 rpm. At the beginning and end of the incubation period, 1-mL aliquots were centrifuged at 9,000×*g* for 10 min at 4°C, and the resultant supernatants were analyzed for ZEN concentration.

### Effect of pH on the ZEN removal ability of CK1

The heat-treated CK1 cells were prepared and harvested as described above. Then, the heat-treated CK1 cells were added to 50 mL aliquots of citrate-phosphate buffer (0.1 M, pH 2.5 or 4.0) or PBS (0.1 M, pH 6.0, 7.0, or 8.0) containing 5 μg mL^-1^ of ZEN to yield a final concentration of 1 × 10^10^ CFU mL^-1^ of bacterial cells. The mixtures were incubated at 37°C for 24 h on an orbital shaker at 120 rpm. At the beginning and end of the incubation period, aliquots of 1 mL were taken and centrifuged at 9,000×*g* for 10 min at 4°C, and the resultant supernatants were analyzed for ZEN concentration.

### Effect of temperature on the ZEN removal ability of CK1

Before the experiments, the temperature of PBS was adjusted to 4, 25, 37, or 42°C. The heat-treated CK1 was prepared as described above and added to 5-mL aliquots of PBS (0.1 M, pH 7.0) containing 5 μg mL^-1^ of ZEN to yield a final concentration of 1 × 10^10^ CFU mL^-1^ of bacterial cells. The mixtures were incubated at 4, 25, 37, or 42°C for 24 h on an orbital shaker at 120 rpm. At the beginning and end of the incubation period, aliquots of 1 mL were taken and centrifuged at 9,000×*g* for 10 min at 4°C, and the resultant supernatants were analyzed for ZEN concentration.

### ZEN adsorption stability of CK1

The ZEN adsorption stability of CK1 was evaluated according to the methods described by El-Nezami et al. [29]. The heat-treated CK1 cells were prepared and harvested as described above. Then, the heat-treated CK1 cells were added to 6-mL of aliquots PBS (0.1 M, pH 7.0) containing 5 μg mL^-1^ of ZEN to yield a final concentration of 1 × 10^10^ CFU mL^-1^ of bacterial cells. After incubation at 37°C for 24 h on an orbital shaker at 120 rpm, a 1-mL aliquot was taken and centrifuged at 9,000×*g* for 10 min at 4°C to harvest the cells for the analysis of the amount of ZEN adsorbed by the CK1 cells. The rest of the aliquot (5.0 mL) was centrifuged at 9,000×*g* for 10 min at 4°C to harvest the remaining cells, and the obtained cells were transferred into a fresh 5-mL of aliquot PBS (0.1 M, pH 7.0) without ZEN. The cells were vortexed for 15 sec, and incubated at 37°C for 5 min on an orbital shaker at 120 rpm for the first round of washing. Then, a 1-mL aliquot was taken and centrifuged at 9,000×*g* for 10 min at 4°C to harvest the cells for analysis of the amount of ZEN adsorbed by the CK1 cells. The ZEN bound by the CK1 cells was obtained by extraction with 1 mL of methanol, and then the quantity of ZEN was determined by HPLC. The rest of the aliquot was centrifuged to harvest the cells, which were then used in the seconded round of washing, by placing the recovered cells in a fresh PBS solution and repeating the washing procedure. The adsorption stability of CK1 to ZEN was determined for five consecutive rounds of washing procedures.

### Determination of ZEN concentration by HPLC

The ZEN concentrations in the samples were analyzed by HPLC based on the method described by Bueno and Oliver [37]. Before the HPLC analysis, the samples were extracted with methanol for 90 min at 180 rpm on an orbital shaker, filtered through a 0.20-μm GHP Acrodisc® syringe filter (Pall Life Sciences, Ann Arbor, MI, USA), and 20 μL was injected into the HPLC to quantify the ZEN in each sample. HPLC analysis was performed using a Shimadzu LC-20 AT delivery system equipped with a RF-10AXL fluorescence detector (Shimadzu Corp., Kyoto, Japan), a Cosmosil 5C18-ARII column (Nacalai Tesque Inc., Kyoto, Japan; 250 × 4.6 mm i.d., particle size 5 μm), and a SIL-10A autoinjector (Shimadzu). The mobile phase was a methanol-water-acetonitrile solution (55:35:10, v/v/v) containing ammonium acetate (15 mM), which had been filtered through a membrane (0.45 μm) and degassed for 5 min before use. The mobile phase flow rate was adjusted to 1 mL min^-1^, and the detection was performed at 365 nm (excitation) and 418 nm (emission). The linearity of the method was verified by analyzing six standard solutions in the range of 0–5 μg mL^-1^ for ZEN, and the ZEN levels in the samples were calculated by comparing the area of the chromatographic peak of the sample with those of the standard curve. The ZEN removal percentage was calculated using the formula: 1-(peak area of ZEN in the supernatant /peak area of ZEN in the positive control) × 100%.

### Statistical analysis

The Statistical Analysis System software package version 13.1 (SAS Institute Inc., Cary, NC, USA) was used to analyze the results. One-way analysis of variance (one-way ANOVA) followed by Duncan’s multiple range test was used to detect the differences between the means of the treatments, and a *p*-value less than 0.05 was considered significant. Each experiment was conducted in triplicate and all results were expressed as means ± standard deviation.

## Results

### Probiotic characteristics of CK1

**Fig 1:**
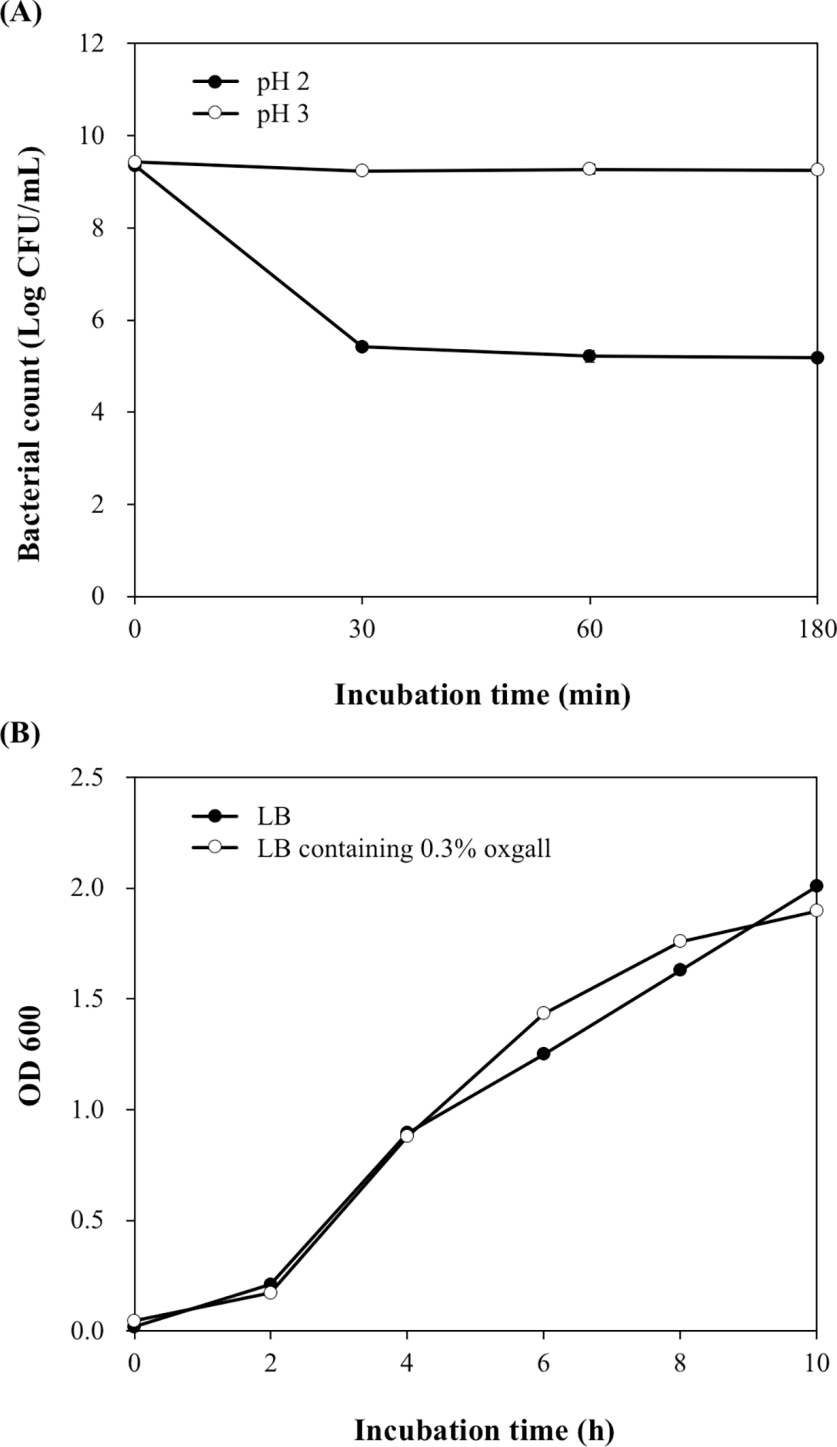
Acidic and bile salt tolerance of *Bacillus licheniformis* CK1. (A) Survival of CK1 after incubation at pH 2.0 or 3.0. (B) Growth curves of CK1 in the absence or presence of 0.3% oxgall.

The probiotic characteristics of CK1, including acidic tolerance, bile salt tolerance, adhesion capability, and anti-pathogenic activities, were evaluated. The acid tolerance of CK1 was examined by culturing the bacterial cells in PBS at pH 2.0 or 3.0 for 3 h, and the bile-salt tolerance of CK1 was determined by culturing the bacterial cells in LB broth containing 0.3% oxgall for 10 h. After incubation at pH 2.0 or 3.0 for 3 h, the bacterial counts of CK1 were 5.18 ± 0.03 and 9.26 ± 0.05 log CFU mL^-1^, respectively (Fig 1A). After incubation in LB broth containing 0.3% oxgall for 10 h, the bacterial concentrations of CK1 were not significantly different from that cultured in LB broth without oxgall (Fig 1B). These results indicated that CK1 has the ability to tolerate acid and bile salts.

**Fig 2:**
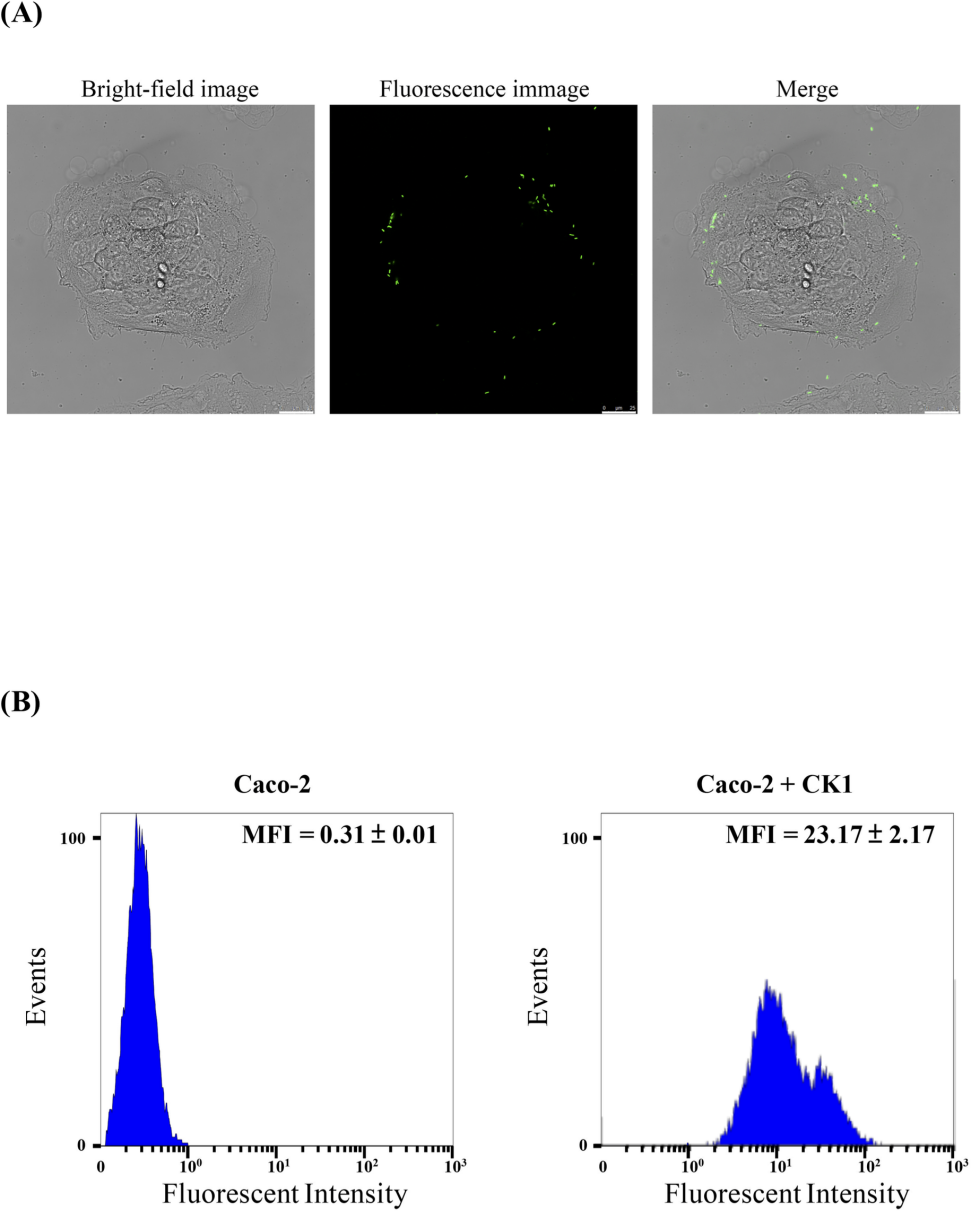
Confocal immunofluorescence microscopic examination and flow cytometric analysis of adhesion capability of *Bacillus licheniformis* CK1 to Caco-2 cells. (A) Confocal microscopic observation of the Caco-2 cells with adherent 5(6)-carboxyfluorescein diacetate N-succinimidyl ester (CFSE)-labeled CK1. (B) Flow cytometric analysis of the green fluorescence intensity of the Caco-2 cells with adherent CFSE-labeled CK1. For each experiment, 10,000 Caco-2 cells were analyzed. The green fluorescence intensity of the Caco-2 cells with adherent CK1 was measured by flow cytometry at 525 nm emission wavelengths. Mean fluorescence intensity (MFI) was calculated from two independent experiments performed in triplicate.

The adhesion capability of CK1 was determined by culturing the CFSE-stained CK1 cells with Caco-2 cells. As shown in Fig 2A, confocal immunofluorescence microscopic observation demonstrated that CK1 could adhere to Caco-2 cells. The adherence of CK1 to Caco-2 cells was further confirmed by flow cytometric analysis. The Caco-2 cells had weak autofluorescence, whereas the Caco-2 cells incubated with CFSE-stained CK1 for 2 h shifted along the fluorescence axis in the histogram plotted in Fig 2B, indicating adhesion of CK1 cells to Caco-2 cells.

The anti-pathogenic activity of CK1 was evaluated by determining the formation of inhibition zone against *B*. *cereus* ATCC 11778, *E*. *coli* O157:H7 ATCC 35150, *L*. *monocytogenes* BCRC 15338, and *S*. *enterica* subsp. *enterica* BCRC 12947. As shown in Table 1, CK1 inhibited the growth of *E*. *coli* O157:H7 ATCC 35150 and *L*. *monocytogenes* BCRC 15338 but did not show inhibition effect on the growth of *B*. *cereus* ATCC 11778 and *S*. *enterica* subsp. *enterica* BCRC 12947.

**Table 1:** Anti-pathogenic activities of *Bacillus licheniformis* CK1.

Indicator strain	Anti-pathogenic activity (Inhibition zone in mm)
*Bacillus cereus* ATCC 11778	NI^a^
*Escherichia coli* O157:H7 ATCC 35150	18.60 ± 0.08^b^
*Listeria monocytogenes* BCRC 15338	16.31 ± 0.01
*Salmonella enterica* subsp. *enterica* BCRC 12947	NI

^a^NI: no inhibition.

^b^Values are the mean ± standard deviations of triplicate measurements.

### Comparison of the ZEN removal abilities of CK1, HSCAS, and LGG

The ZEN removal abilities of CK1, HSCAS, and LGG were compared with each other in PBS (0.1 M, pH 7.0) containing 5 μg mL^-1^ of ZEN with a final HSCAS concentration of 1% (w/v) or a bacterial concentration of 1 × 10^10^ CFU mL^-1^. As shown in Table 2, the ZEN removal percentages of HSCAS, viable LGG, and viable CK1 were 45.9 ± 0.1%, 48.4 ± 2.4%, and 73.0 ± 1.8%, respectively, indicating that the ZEN removal ability of CK1 is significantly higher than that of HSCAS and LGG.

**Table 2:** ZEN removal abilities of hydrated sodium calcium aluminosilicate (HSCAS), *Lactobacillus rhamnosus* GG (LGG), and *Bacillus licheniformis* CK1 (CK1).

Sample*	ZEN removal percentage (%)
HSCAS	45.9 ± 0.1^a^
Viable LGG	48.4 ± 2.4^a^
Viable CK1	73.0 ± 1.8^b^
Acid-treated CK1	71.0 ± 0.5^b^
Heat-treated CK1	74.3 ± 1.8^b^

*HSCAS concentration = 1% (w/v); LGG and CK1 concentrations = 1 × 10^10^ CFU mL^-1^.

^a, b^Means with different superscripts are significantly different (*p* < 0.05).

CK1 cells were subjected to acid or heat treatments, and then the ZEN adsorption abilities of acid- and heat-treated cells were compared to that of viable CK1 cells. As shown in Table 2, the ZEN binding percentage of acid- and heat-treated CK1 were 71.0 ± 0.5% and 74.3 ± 1.8%, respectively, which were not different from that of viable CK1.

### Effects of ZEN concentrations on the ZEN removal ability of CK1

**Fig 3:**
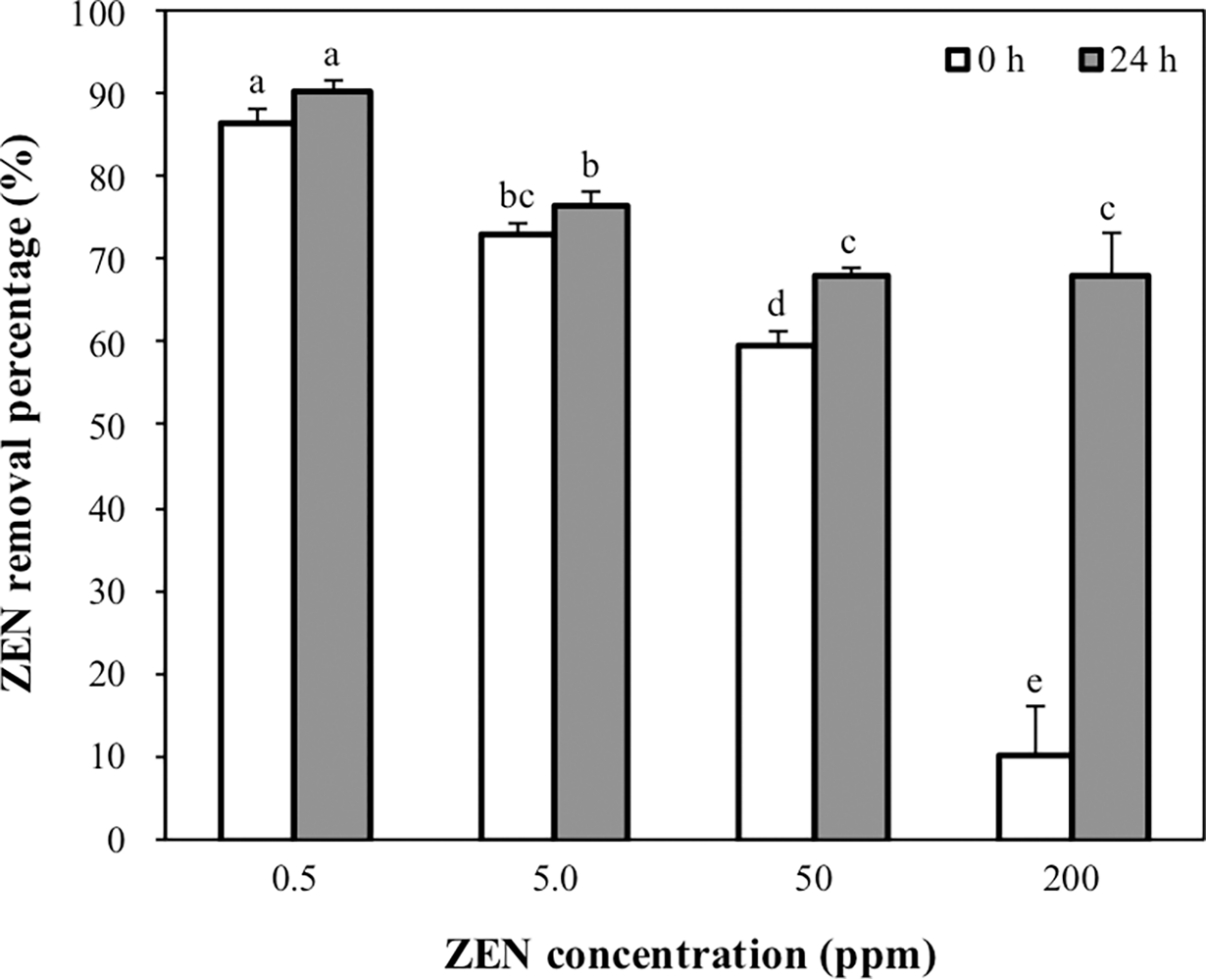
Effects of zearalenone (ZEN) concentrations on the ZEN removal ability of *Bacillus licheniformis* CK1. The heat-treated CK1 cells (1 × 10^10^ CFU mL^-1^) were incubated with different concentrations of ZEN (0.5, 5, 50, and 200 μg mL^-1^, pH 7.0) at 37°C for 24 h. The ZEN removal percentage was determined at the beginning and end of incubation. Bars with different letters indicate significant difference (*p* < 0.05).

Effects of ZEN concentrations on the ZEN removal ability of CK1 were evaluated by determining the ZEN removal percentage of the heat-treated CK1 (1 × 10^10^ CFU mL^-1^) in PBS containing ZEN concentrations from 0.5 to 200 μg mL^-1^. At low levels of ZEN (0.5 and 5 μg mL^-1^), more than 70% of ZEN was removed by CK1 immediately after mixing with the ZEN (Fig 3). At high concentrations of ZEN (50 and 200 μg mL^-1^), 60% and 10%, respectively, of the ZEN was removed by CK1 immediately after mixing with the ZEN. After 24 h of incubation, the amounts of ZEN removed by the CK1 cells were not increased at low concentrations of ZEN (0.5 and 5 μg mL^-1^). However, the amounts of ZEN removed by the CK1 cells increased significantly at the high levels of ZEN (50 and 200 μg mL^-1^) after 24 h of incubation (Fig 3).

### Effect of pH on the ZEN removal ability of CK1

**Fig 4:**
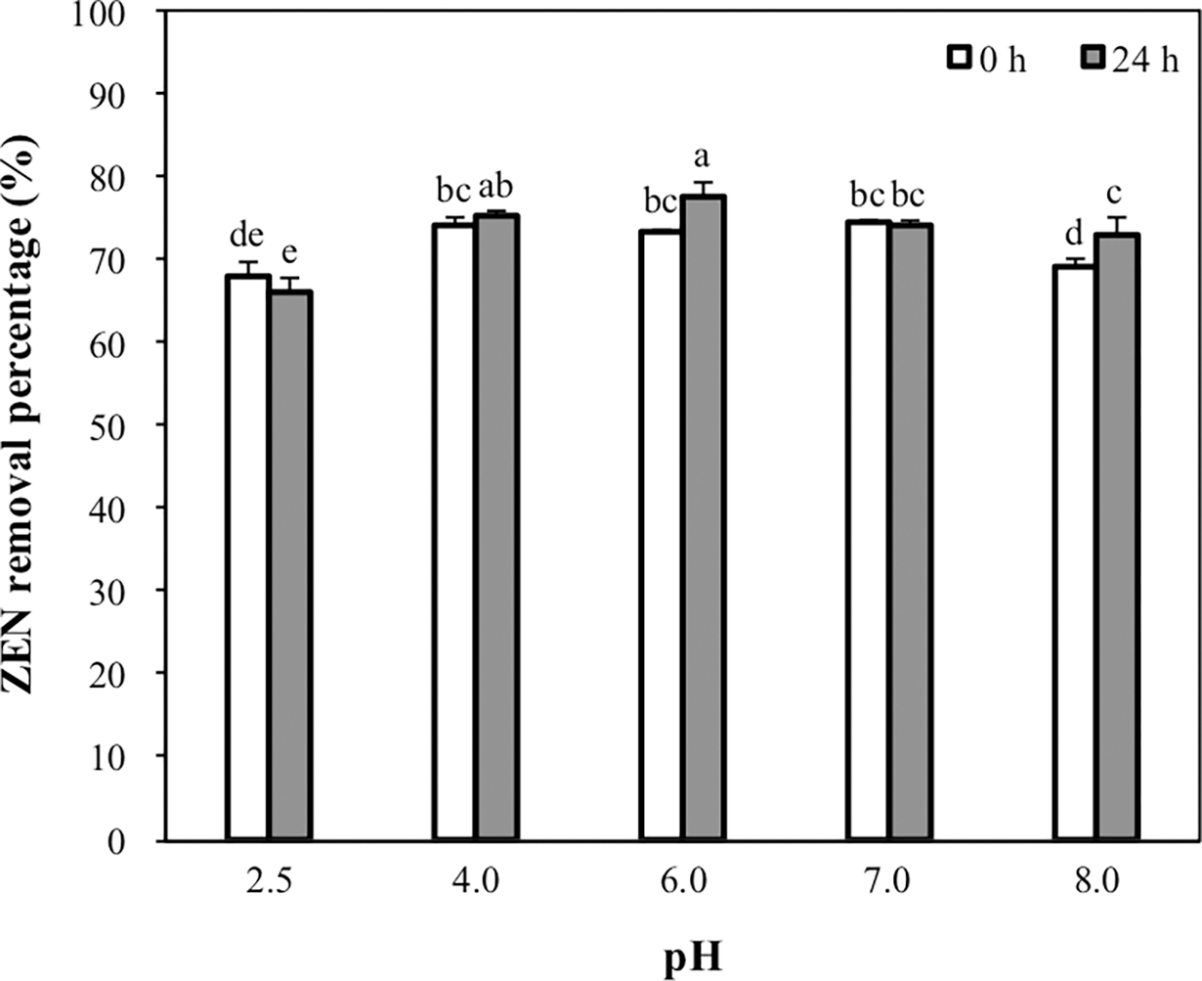
Effects of pH on the zearalenone (ZEN) removal ability of *Bacillus licheniformis* CK1. The heat-treated CK1 cells (1 × 10^10^ CFU mL^-1^) were incubated with 5 μg mL^-1^ of ZEN at pH 2.5, 4.0, 6.0, 7.0, or 8.0 at 37°C for 24 h. The ZEN removal percentages were determined at the beginning and end of incubation. Bars with different letters indicate significant difference (*p* < 0.05).

The effect of pH on the ZEN removal ability of CK1 was determined immediately after mixing or after 24 h of incubation of CK1 with ZEN at pH 2.5, 4.0, 6.0, 7.0, or 8.0. Immediately after the addition of ZEN, the quantities of ZEN removed by CK1 at pH 2.5 and 8.0 (67.7% and 69.2%, respectively) were significantly lower than those at pH 4.0, 6.0, and 7.0 (74.0%, 73.1%, and 74.2%, respectively) (Fig 4). After 24 h of incubation at pH 6.0 and 8.0, the amounts of ZEN removed by CK1 had increased significantly (Fig 4).

### Effect of temperature on the ZEN removal ability of CK1

**Fig 5:**
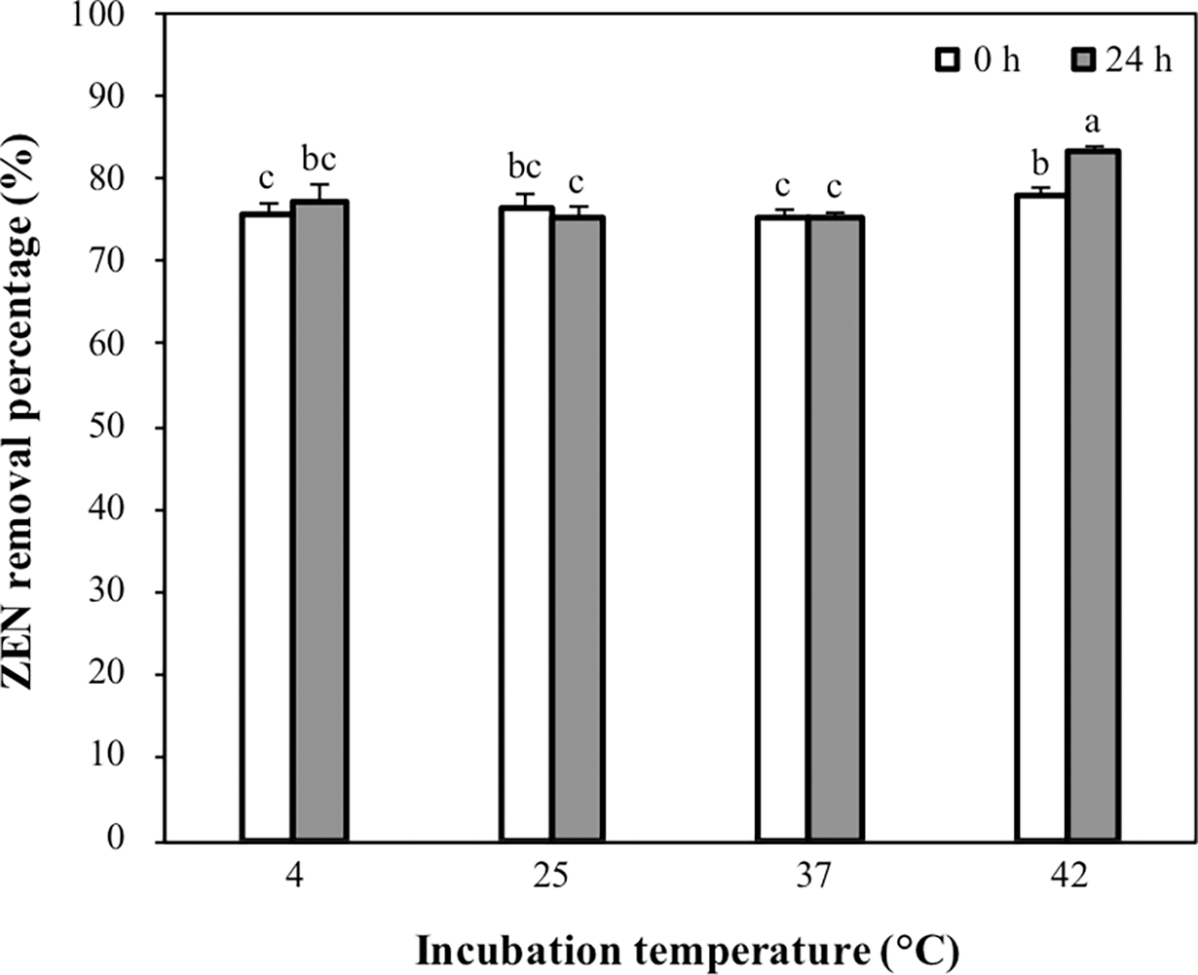
Effects of incubation temperature on the zearalenone (ZEN) removal ability of *Bacillus licheniformis* CK1. The heat-treated CK1 cells (1 × 10^10^ CFU mL^-1^) were incubated with 5 μg mL^-1^ of ZEN (pH 7.0) at 4, 25, 37, or 42°C for 24 h. The ZEN removal percentages were determined at the beginning and end of incubation. Bars with different letters indicate significant difference (*p* < 0.05).

The effect of temperature on the ZEN removal ability of CK1 was determined immediately after adding or after 24 h of incubation of CK1 with ZEN at 4, 25, 37, or 42°C. More than 75% of ZEN was removed by CK1 immediately after coming into contact with ZEN, and CK1 showed the highest ZEN removal ability at 42°C (Fig 5). After 24 h of incubation at 42°C, the amounts of ZEN removed by CK1 increased significantly (Fig 5).

### ZEN adsorption stability of CK1

**Fig 6:**
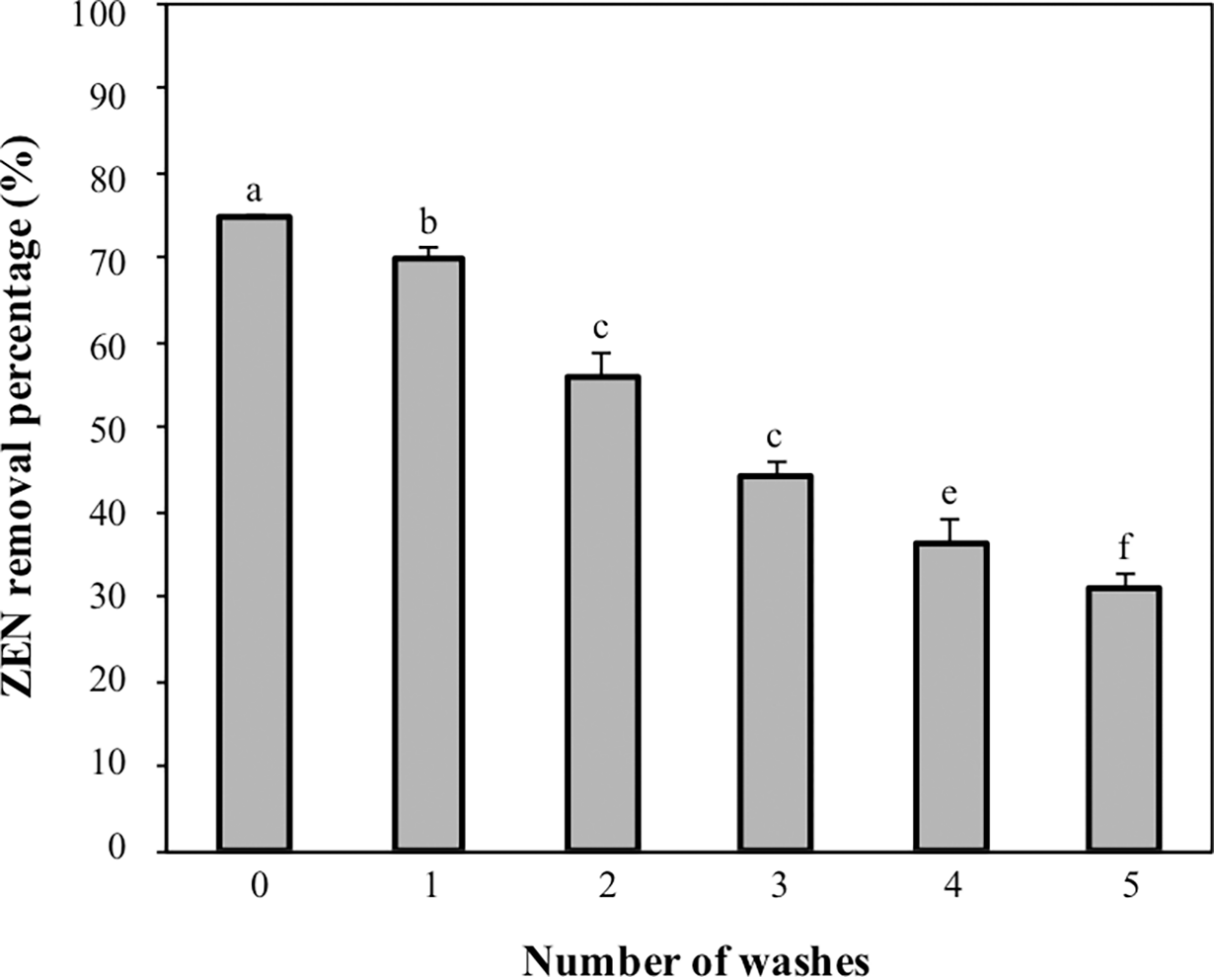
Zearalenone (ZEN) adsorption stability of *Bacillus licheniformis* CK1. Bars with different letters indicate significant difference (*p* < 0.05).

The ZEN adsorption stability of CK1 was evaluated by determining the amounts of ZEN bound by CK1 during five consecutive rounds of washing procedures. Fig 6 shows that the amounts of ZEN bound by CK1 decreased with the increasing rounds of washing procedures. After five rounds of washing, the amount of ZEN bound by CK1 was 31.0 ± 1.7%.

## Discussion

According to the recommendation of FAO, probiotic potential strains must possess acid and bile salt tolerant ability, adherence capability, and anti-pathogenic activities [38]. The acidic conditions of the stomach and the bile salts in the duodenum are the most difficult hurdles associated with the survival of the probiotics in the gastrointestinal tract. In this study, CK1 survived after incubation at pH 2.0 or 3.0 for 3 h and grew well in LB broth containing 0.3% oxgall (Fig 1), indicating that CK1 might be able to survive transit through the stomach and survive in the intestinal tract where it can exert its beneficial effects.

After transit through the stomach and surviving in the intestinal tract, probiotic potential strains must be able to adhere to the intestinal mucosa to extend their residence time in the intestinal tract and hence allows them to achieve their maximum probiotic effects. Moreover, probiotics possess the ability to adhere to intestinal mucosa can provide competition against the adhesion of pathogens to the intestinal epithelial cells [39,40]. In this study, Caco-2 cells incubated with fluorescent-labeled CK1 showed a shift along the fluorescence axis when compared with the autofluorescence expressed by Caco-2 cells alone (Fig 2), indicating that CK1 possessed adherence capability to Caco-2 cells.

Another important property of probiotic potential strains is their anti-pathogenic activity [38] As shown in Table 1, CK1 inhibited the growth of *E*. *coli* O157:H7 ATCC 35150 and *L*. *monocytogenes* BCRC 15338. Live animals are the main source of contamination, and may transmit *E*. *coli* O157 and *L*. *monocytogenes* to humans through the consumption of contaminated foods [41,42]. Therefore, a reduction of the carrier rate of *E*. *coli* O157 and *L*. *monocytogenes* in cattle and pigs at the herd level could reduce contamination at the slaughterhouse and subsequently the load introduced into the food chain [42,43]. In the present study, we found that CK1 inhibited the growth of *E*. *coli* O157: H7 and *L*. *monocytogenes*, which suggested that CK1 has the potential to be used for prevention and control of these two pathogens.

ZEN is one of the most harmful mycotoxins in animal feed and causes reproductive disorders in farm animals, resulting in significant economic losses due to the lower efficacy of livestock production [1,3]. The decrease of ZEN bioavailability by mycotoxin-detoxifying agents is one of the most commonly used strategies for decontamination of ZEN in feeds [1]. In this study, we compared the ZEN removal abilities of CK1 with the biological mycotoxin-adsorbing agents LGG and the mineral mycotoxin-adsorbing agent HSCAS. We found that the ZEN removal ability of the biological mycotoxin-adsorbing agent LGG was similar to that of the mineral mycotoxin-adsorbing agent HSCAS but significantly lower than that of CK1 (Table 2). Thus, CK1 was the best among these three ZEN-detoxifying agents and has the potential to be used as feed additive to decrease the bioavailability of ZEN.

The structure of ZEN contains a diphenolic group makes it much more hydrophobic than aflatoxins. Therefore, the dominant mechanism of binding of a mycotoxin-adsorbing agent to ZEN will probably be through hydrophobic interactions [44]. Natural clays like aluminosilicates appeared to have little adsorption ability against ZEN [45]. However, the ZEN adsorption efficacy of clays can be enhanced by physical or chemical modifications, such as nanonization of particles to increase their surface area and porosity or alteration of the surface properties by exchange of structural charge-balance cations with high molecular weight quaternary amines to increase the hydrophobicity [44]. HSCAS is characterized as a good aflatoxin-adsorbing agent, but its efficacy against other mycotoxins is very limited [27]. In this study, we found that HSCAS removed 45.9% of ZEN, indicating that a heating processed could increase the ZEN removal ability of aluminosilicates.

The cell wall compositions of Gram-positive bacteria, such as *Bacillus* and *Lactobacillus*, are thick layers of peptidoglycans, teichoic acids, polysaccharides, and surface proteins [46,47]. The peptidoglycans extracted from *B*. *subtilis* possess fumonisin B1 adsorption ability [48], while the polysaccharide components of the cell wall of LGG possess ZEN adsorption ability [15]. The thickness of the peptidoglycan structure may be reduced, and its pore size may be increased via heat and acid treatments. The amide linkages in the structure of peptidoglycans are broken by acid treatment. The glycosidic linkages of the polysaccharide may be broken via the Maillard reaction, which occurs between polysaccharides, peptides, and proteins after heat treatments. Surface proteins may be denatured by heat or acid treatment [29]. Therefore, the adsorption abilities of the biological mycotoxin-adsorption agents are affected by acid or heat treatments [15,29,49,50]. In a previous study, the acid- or heat-treated *B*. *natto* CICC24640 showed a lower ZEN removal ability than the untreated analog [49]. However, we found that the ZEN removal ability of CK1 was not affected by acid or heat treatment. We suggested that the compounds that contribute to ZEN removal ability of CK1 are acid and heat resistant; thus, the ZEN removal ability of CK1 might not be changed when it enters the acidic gastric environment or is exposed to heat during the pelleting process.

Removal of ZEN by CK1 is a rapid reaction since more than 70% of ZEN was removed by CK1 immediately after coming into contact with ZEN at low initial concentrations of ZEN (0.5 and 5 μg mL^-1^). At high initial concentrations of ZEN (50 and 200 μg mL^-1^), lower proportions of ZEN were removed by CK1 cells (60% and 10%, respectively) immediately after the addition ZEN. Nonetheless, the quantity of ZEN removed by the CK1 cells increased to about 60% after 24 h of incubation (Fig 3). These results indicate that the amounts of ZEN removed by CK1 did not increase with incubation time at lower initial ZEN concentrations (0.5 and 5 μg mL^-1^) but did increase with incubation time at high initial ZEN levels (50 and 200 μg mL^-1^). We suggested that the increase in incubation time gives free ZEN greater opportunity to be bound to remaining binding sites on the surface of CK1 when ZEN is at higher concentrations.

After ingestion by mouth, feed digesta passes through various pH conditions in the gastrointestinal tract of the animal. In the gastrointestinal tract of swine fed ad libitum, the average pH in the stomach, small intestinal, caecum, and colon are 4.4, 6.1–6.7, 6.0–6.4, and 6.1–6.6, respectively [51]. Therefore, the removal ability of mycotoxin-detoxifying agents must be evaluated under different pH conditions. We evaluated the ZEN removal ability of CK1 immediately after addition and after 24 h of incubation of CK1 with ZEN at pH 2.5, 4.0, 6.0, 7.0, and 8.0. We found that more than 65% of ZEN was removed by CK1 at the pH values ranging from 2.5–8.0, regardless of the time after adding the ZEN (immediately after or after 24 h of incubation) (Fig 4). In addition, CK1 removed the largest amounts of ZEN at pH 6.0 than at the other pH values tested. According to these results, we suggested that CK1 could remove ZEN efficiently in the pH environment of the gastrointestinal tract of animals.

The evaluation of temperature on the ZEN removal ability of CK1 was tested at four different temperatures: 4, 25, 37, and 42°C. Immediately after coming into contact with ZEN, the amounts of ZEN removed by CK1 at all of the temperatures were similar. However, the quantities of ZEN removed by CK1 increased significantly after incubation at 42°C for 24 h but not after incubation at 4, 25, or 37°C for 24 h (Fig 5). Adsorption processes are classified as either physical adsorption (van der Walls adsorption) or chemisorption (activated adsorption). Physical adsorption usually occurs at low temperatures, while chemical adsorption can occur at high temperature. Physical adsorption, rather than chemical adsorption, may play a more important role in the mycotoxin adsorption reaction of the biological mycotoxin-adsorbing agents [44]. Most of the biological mycotoxin-adsorbing agents adsorb mycotoxins rapidly at temperatures in the range of 4 to 37°C, and the hydrophobic pockets on the bacterial surface may be contributed to the mycotoxin adsorption [15]. In this study, more than 75% of ZEN was removed by CK1 immediately after coming into contact with ZEN at 4, 25, 37, or 42°C, indicating CK1 removed ZEN rapidly at low temperatures. Furthermore, these results suggest that the removal of ZEN by CK1 may be through a mechanism similar to that of other biological mycotoxin-adsorbing agents.

The mechanism of action of the mycotoxin-adsorbing agents is through the formation of adsorbing agent-mycotoxin complexes, which then pass through the animal’s gastrointestinal tract to be eliminated in the faeces. Therefore, mycotoxin-adsorbing agents should be able to bind the mycotoxins stably without dissociating in the digestive tract of the animal. The binding stability of mycotoxin-adsorbing agents could be evaluated by washing the adsorbing agent-mycotoxin complexes with several rounds of washing procedures [29], although there is no washing procedure in practical application of mycotoxin-adsorbing agents. In this study, we evaluated the binding stability of CK1 to ZEN by determining the amounts of ZEN bound by CK1 during five consecutive rounds of washing procedures. After five rounds of washing, the CK1 cells retained more than 30% of their initially bound ZEN (Fig 6). Since ZEN is adsorbed by CK1 through physical adsorption to hydrophobic pockets on the bacterial cell surface, the ZEN adsorbed by CK1 could be dissociated from the ZEN-CK1 complex during the washing procedures. In a previous study, almost all of the aflatoxin B1 dissociated from its complex with *L*. *plantarum* PTCC1058 after three rounds of washing [52]. In another study, almost all the aflatoxin B1 adsorbed by *L*. *amylovarus* CSCC 5160 was dissociated, while *L*. *amylovarus* CSCC 5159 and *L*. *rhamanosus* Lc1/3 retained 17.4% and 32.2%, respectively, of their initial bound aflatoxin B1 [53]. These results suggested that the stabilities of the complexes formed between bacteria and mycotoxins are microbe-specific. Our data show that even after five rounds of washing, CK1 cells retained more than 30% of their initially bound ZEN, indicating that the binding of CK1 to ZEN was stable and could provide protection against ZEN.

Following a thorough review of the relevant literature, only a few studies on the bio-removal of ZEN by *Bacillus* spp. have been reported, and the strains in these studies include *B*. *subtilis* 168, *B*. *natto CICC 24640* [49], *B*. *amyloliquefaciens* ZDS-1 [54], and *B*. *amyloliquefaciens* LN [55]. Among these ZEN-removal *Bacillus* strains, only *B*. *amyloliquefaciens* LN has been studied for its safety and probiotic properties [55]. *B*. *amyloliquefaciens* LN was non-hemolytic and non-enterotoxin producing, displayed probiotic characteristics including acidic tolerance, bile salt tolerance, and anti-pathogenic activities, and showed an efficient ZEN removal ability [55]. However, the adherence capability of *B*. *amyloliquefaciens* LN to epithelial cells and the production of fibrolytic enzymes and protease by *B*. *amyloliquefaciens* LN have not been demonstrated. In a previous study, we found that CK1 displayed high levels of extracellular xylanase, cellulase, and protease activities [31]. In the present study, we demonstrated that CK1 possessed adherence capability to Caco-2 cells. Moreover, in comparison with *B*. *amyloliquefaciens* LN, CK1 seems to have a higher ZEN removal ability. Immediately after adding of bacterial cells to the PBS containing 5 μg mL^-1^ of ZEN, the ZEN removal percentages of *B*. *amyloliquefaciens* LN was 34.4% [55], while the ZEN removal percentages of CK1 was 73.0% (Table 2). These findings suggest that CK1 has a great potential for the development of feed additives to remove ZEN.

## Conclusions

CK1 displayed probiotic characteristics including acidic tolerance, bile salt tolerance, adherence capability, and anti-pathogenic activities. CK1 removed more than 70% of ZEN at 37°C and pH 7.0 and showed a better ZEN adsorption ability than LGG and HSCAS. At a pH range of 2.5–8.0 or temperatures between 4 and 42°C, CK1 removed more than 65% of ZEN. After five consecutive rounds of washing procedures, CK1 retained more than 30% of its initially bound ZEN. These results indicated that CK1 removed ZEN effectively. Thus, we suggested that CK1 has a great potential for the development of feed additive to remove ZEN.
